# Stroke Scales as Assessment Tools in Emergency Settings: A Narrative Review

**DOI:** 10.3390/medicina58111541

**Published:** 2022-10-27

**Authors:** Hrvoje Budinčević, Andrija Meštrović, Vida Demarin

**Affiliations:** 1 Department of Neurology, Sveti Duh University Hospital, 10000 Zagreb, Croatia; 2 Department of Neurology and Neurosurgery, Faculty of Medicine, J.J. Strossmayer University of Osijek, 31000 Osijek, Croatia; 3 International Institute for Brain Health, 10000 Zagreb, Croatia

**Keywords:** stroke, stroke scales, acute stroke

## Abstract

In the last 20 years, substantial improvements have been made in stroke recanalization treatment. Good outcomes after modern reperfusion treatment require the rapid and accurate identification of stroke patients. Several stroke rating scales are available or have been proposed for the early recognition of stroke and the evaluation of stroke severity and outcome. This review aims to provide an overview of commonly used stroke scales in emergency and clinical settings. The most commonly used scale in a prehospital setting for stroke recognition is the Face, Arms, Speech, Time (FAST) test. Among many prehospital stroke scales, the Los Angeles Prehospital Stroke Screen has the highest sensitivity and specificity for confirming stroke diagnosis. The National Institutes of Health Stroke Scale (NIHSS) is the most recommended tool for the evaluation of stroke patients in hospital settings and research, and it has two variants: the shortened NIHSS for Emergency Medical Service and the modified NIHSS. The evaluation of comatose patients usually involves assessment with the Glasgow Coma Scale, which is very useful in patients with hemorrhagic stroke or traumatic brain injury. In patients with subarachnoid hemorrhage, the outcome is usually accessed with the Hunt and Hess scale. A commonly used tool for stroke outcome evaluation in clinical/hospital settings and research is the modified Rankin scale. The tools for disability evaluation are the Barthel Index and Functional Independence Measure.

## 1. Introduction

Stroke is associated with high rates of disability and mortality worldwide [1]. Despite primary and secondary stroke prevention measures, the global burden of stroke is still high, and stroke epidemiological data show that it is the second leading cause of death in the world, after ischemic heart disease [2]. The absolute numbers of stroke substantially increased from 1990 to 2019, with 70.0% increase in incident strokes, 43.0% increase in deaths from stroke, 102.0% increase in prevalent strokes, and 143.0% increase in disability-adjusted life years (DALYs) [3]. The vast majority of the global stroke burden (86.0% of deaths and 89.0% of DALYs) were in lower-income and lower–middle-income countries [3]. This clinical disorder is characterized by a sudden onset of a focal neurological deficit [4]. In the last 20 years, reperfusion therapy using intravenous thrombolysis and/or mechanical thrombectomy has dramatically increased the percentage of good functional outcomes in these patients [5]. Good outcomes after modern reperfusion therapy require a rapid and accurate identification of stroke patients [5]. For better clinical outcomes, public awareness and emergency prehospital and hospital care are crucial [6]. The rapid and accurate identification and quantification of stroke may be possible using a stroke scale [7]. However, in 30% of cases, the usage of stroke scales in prehospital settings does not adequately recognize stroke [8]. Some prehospital stroke scales have been developed for the recognition of large vessel occlusion strokes, similar to stroke recognition, but about 20% of strokes due to large vessel occlusion still remain unrecognized by these scales [9]. Nevertheless, the outcome of stroke may be predicted using some of the rating scales, for example, the National Institutes of Health Stroke Scale (NIHSS), which has shown a reliable correlation with good clinical outcomes if the score is less than 4 in the first 48 h [10]. On the other hand, some scales might be useful for determining eligibility criteria; for example, patients with moderate disability before recurrent stroke with a modified Rankin score ≥2 are not eligible candidates for mechanical thrombectomy according to American Heart Association/American Stroke Association (AHA/ASA) guidelines [11].

This review aims to provide an overview of commonly used stroke scales in emergency, clinical prehospital and hospital settings, and research.

## 2. Prehospital Stroke Scales

There are several stroke screening scales that have been created for stroke recognition within the population. The most commonly used tool is the Face, Arms, Speech, Time (FAST) test, especially in emergency settings [12]. Despite FAST being very useful for anterior circulation strokes, it can miss over 70% of patients with posterior circulation strokes [13]. A useful tool for stroke screening is the Gaze, Face, Arms, Speech, Time (G-FAST) test, in which the gaze evaluation is also included [14]. For a better diagnosis of posterior circulation strokes, it can be helpful to also assess balance (B) and eye (E) symptoms in FAST, known as BE-FAST [12]. Another modification of the FAST scale is the FAST-ED scale, which includes eye deviation and anosognosia/neglect [15]. The FAST-ED scale has a higher predictive value for strokes related to large vessel occlusion and eligible candidates for mechanical revascularization (thrombectomy) [15]. Currently, in prehospital settings, there are several stroke scales that can guide emergency medical staff in triaging patients with acute stroke [16]. Commonly used scales are the following: (1) the 3-item stroke scale (3I-SS), (2) the Austrian Prehospital Stroke Scale (APSS), (3) the Cincinnati Prehospital Stroke Scale (CPSS), (4) the Los Angeles Stroke Screen (LAPSS), (5) the Rapid Arterial Occlusion Evaluation (RACE) scale, and (6) the shortened NIHSS for EMS (sNIHSS-EMS) [16,17,18]. In recent years, there has been more focus on the identification of large vessel occlusion (LVO) strokes, which are candidates for mechanical revascularization procedures [7]. However, none of the available scales has optimal accuracy in the prediction of this type of stroke, and some mild or LVO strokes might be unrecognized [7]. Selected abbreviated prehospital stroke scales are presented in Table 1.

For stroke mimics, there is the Recognition of Stroke in the Emergency Room (ROSIER) scale, which has proved to be better than the FAST, CPSS, and LAPSS scales [17,19]. Another scale, FABS, is a useful screening tool for recognizing stroke mimics [20]. The Telestroke Mimics Score is feasible for differentiating stroke mimics in telestroke networks and consultations [21].

**Table 1:** Selected abbreviated prehospital stroke scales [16,18,22,23].

Scale	Rating System	Sensitivity/Specificity	Considerations/Cut-Off Value
3-Item Stroke Scale (3I-SS)	Level of consciousness (0–2) Gaze and head deviation (0–2) Hemiparesis (0–2)	67%/92%	Large vessel occlusion stroke recognition ≥4
Austrian Prehospital Stroke Scale (APSS)	Facial weakness (0–1) Arm weakness (0–2) Speech (0–2) Leg weakness (0–2) Gaze deviation (0 or 2)	64%/86%	Large vessel occlusion stroke recognition ≥4
Cincinnati Prehospital Stroke Scale (CPSS)	Gaze (2) Arm weakness (1) Level of consciousness (1)	89%/73%	Severe stroke recognition N/A
Field Assessment Stroke Triage for Emergency Destination (FAST-ED)	Facial weakness (0–1) Arm weakness (0–2) Speech changes (0–2) Eye deviation (0–2) Anosognosia/neglect (0–2)	61%/89%	Large vessel occlusion stroke recognition >4
Los Angeles Prehospital Stroke Screen (LAPSS)	(1) Age > 45 years; (2) Seizure/epilepsy history is absent; (3) Symptom duration <24 h; (4) The patient is not a full-time wheelchair user or bedridden; (5) The blood glucose is between 60 and 400 mg/dL; (6) A unilateral deficit is present in one of the three items (arm drift, hand grip, or face).	91%/97%	“In-the-field” stroke diagnosis N/A
Rapid Arterial Occlusion Evaluation (RACE)	Aphasia/agnosia (0–2) Facial weakness (0–2) Arm or leg weakness (0–2) Leg weakness (0–2) Gaze–eye deviation (0–1)	85%/68%	Large vessel occlusion stroke recognition ≥5
Shortened NIHSS for EMS (sNIHSS-EMS)	Level of consciousness (0–3) Facial weakness (0–3) Left motor arm (0–4) Right motor arm (0–4) Left motor leg (0–4) Right motor leg (0–4) Sensory (0–2) Best language (0–3) Dysarthria (0–2)	70%/81%	Large vessel occlusion stroke recognition ≥6

## 3. Commonly Used Scales in Hospital Settings

The National Institutes of Health Stroke Scale (NIHSS) is a broadly adopted stroke impairment and severity scale in hospital settings [24]. It consists of 15 evaluating segments that are used to estimate and measure stroke severity, with a maximum score of 42 points [25,26]. The NIHSS may be performed quickly and can predict neurological short-term and long-term outcomes [27,28]. It is also feasible for trained healthcare providers without expertise in neurology [29]. It was originally developed in 1989 and is now widely used for outcome measures [28].

However, the NIHSS has some limitations: (1) it does not evaluate the cranial nerves in detail; (2) it underestimates the severity of disease in patients with brainstem or cerebellar infarction; (3) some discrete neurological deficits might be missed; (4) it does not accurately reflect the stroke severity of each cerebral hemisphere; (5) the least reliable score is present in patients with cognitive dysfunction; (6) some clinical changes in repeated examinations might not be shown as changes on the scale; and (7) an abnormality on the NIHSS does not support or refute a stroke diagnosis [24,30,31,32].

Despite the standard full version of the NIHSS, there are other versions designed for an emergency setting, the most promising of which are: (1) the modified NIHSS and (2) the shortened NIHSS-EMS [32,33,34,35]. All presented NIHSS versions are valid and reliable, and may be used in clinical and research settings [32,33,34,35]. Table 2 shows the NIHSS and the mentioned modifications. For telemedicine use, the NIHSS remains a swift and reliable clinical tool [36]. Moreover, retrospective NIHSS scoring is also possible with the use of a specific algorithm [26]. Contrary to the NIHSS scale, the Stroke Impact Scale (SIP) evaluates the health status in patients with chronic stroke and does not lack association with measures of impairment and functional limitation [37]. The SIS was developed to grade changes in the impairment and functional limitations in the following clinical contexts: (1) hand function, (2) activities of daily living, (3) mobility, (4) emotion, (5) communication, (6) memory, (7) thinking, and (8) participation after stroke [37].

Other popular stroke scales that assess stroke severity are: (1) the Canadian Neurological Scale, (2) the European Stroke Scale, and (3) the Scandinavian Stroke Scale [22]. The scores for the NIHSS and Scandinavian Stroke Scale are easily interconverted with great accuracy [38]. The Canadian Neurological Scale is easier and quicker to perform than the NIHSS [22,39]. Similarly to the NIHSS, it has been validated for retrospective use [22,39]. The European Stroke Scale has been developed for the evaluation of patients with stroke in the middle cerebral artery irrigational territory [40]. Table 2 shows the National Institutes of Health Stroke Scale variants.

The evaluation of comatose patients usually involves assessment with the Glasgow Coma Score (GCS), which is very useful in hemorrhagic stroke or traumatic brain injury patients [41]. The GCS is part of: (1) the World Federation of Neurological Surgeons (WFNS) scale, (2) the ICH score, and (3) the Full Outline of UnResponsiveness (FOUR) score [41,42,43]. The GCS has three clinical parameters to evaluate: eye opening, verbal response, and motor response. The WFNS scale is based on the GCS and the presence of motor deficits [42]. The ICH score might be used for clinical outcome prediction, as it includes the following: GCS, volume of hematoma, appearance and quantity of intraventricular hemorrhage, infratentorial location, and older age [43]. The FOUR score addresses some pitfalls in GCS by including the following stages of evaluation: eye movements, motor examination, reflexes of the brainstem, and pattern of respiration [44,45].

The assessment of patients with subarachnoid hemorrhage (SAH) includes the use of several scales in daily clinical practice. The most commonly used scale is the Hunt and Hess scale for patients with confirmed subarachnoid hemorrhage [46]. The initial score is associated with the severity of SAH [42]. Despite its extensive use, there are some conflicting data regarding its utility for prognosis and interobserver variability [42,47,48]. Fisher and the modified Fisher scale, which include the computed tomography analysis of the brain, predict the risk of delayed cerebral ischemia after SAH with more accuracy [42].

## 4. Other Stroke Scales

The two most popular disability scales are the Barthel Index (BI) and Functional Independence Measure (FIM) [22]. The BI consists of 10 measures that cover essential aspects of self-care and physical dependency, and the FIM measures 13 aspects of motor function and 5 aspects of cognitive function [22]. A commonly used tool for stroke outcome or stroke handicap is the modified Rankin scale [22,49]. There are many more scales that are not covered in this review, such as scales for specific neurological deficits (e.g., Berg Balance Scale, Fugl–Meyer Assessment, Mini Mental State Examination, Montreal Cognitive Assessment, Beck Depression Inventory, Hamilton Depression Scale, and Hachinski vascular dementia scale) and quality-of-life issues. Table 3. shows rating systems for Glasgow Coma Score, Hunt & Hess and modified Rankin Scale [42].

## 5. Future Directions and Limitations

In our opinion, the utilization of some rating scales should become routine practice for the evaluation of patients with stroke. Training for more demanding scales is necessary to reduce inter-rater variability and to increase reliability [50]. Some training for stroke scales is freely available. There is a need for using the same rating scale at least in hospital or, if possible, on a national scale, which could produce a standardized approach, improve communication, and decrease possible misunderstandings. These scales, especially those that are used in hospital settings, may modify clinical outcomes if they are standardized and performed routinely and regularly [50].

Unfortunately, the currently available scales have limitations regarding recognizing some signs and symptoms, mostly related to posterior circulation strokes [51]. In addition, these scales are not able to differentiate among stroke subtypes, possibly affecting clinical decision making. The pathophysiology, prognosis, and clinical features of lacunar strokes are different from other types of ischemic strokes, so differentiating this stroke subtype might be under-recognized, since most scales in prehospital settings are associated with large vessel occlusion strokes [52]. Other limitations of using these scales are proper training requirements and the validation of scales, the need for clear clinical protocols, and defining the times of evaluation [53]. Rating scales that are used in hospital settings are more time relevant than rating scales for prehospital settings, e.g., the NIHSS might be completed in 10 min, and some cognitive assessments take more time [50].

## 6. Limitation of the Review

Our review only includes commonly used scales in routine clinical work and research. The major limitation of the review is that we are not able to include all available scales, especially for specific neurological deficits, which are only listed. Therefore, the caveat is that we do not present the result of a systematic review and meta-analysis, which would provide a more detailed and objective analysis of stroke scales.

## 7. Conclusions

Stroke rating scales are useful tools in everyday clinical practice and research. Despite their limitations, specific scales are used either as stroke recognition tools or as a quantification tool for measuring severity, disability, outcome, or other aspects of stroke. The currently preferred scales are: (1) FAST, for prehospital settings and stroke recognition by the public, and (2) the NIHSS and mRS for clinical in-hospital evaluation and research purposes.

## Figures and Tables

**Table 2:** The National Institutes of Health Stroke Scale variants [16,23,32,33,34,35].

Scale/Evaluation of	NIHSS (0–42)	sNIHSS-EMS (0–29)	mNIHSS (0–31)
1a. Level of Consciousness	0 = Alert; responsive 1 = Not alert; somnolent 2 = Not alert; soporose 3 = Comatose	0 = Alert; responsive 1 = Not alert; somnolent 2 = Not alert; soporose 3 = Comatose	
1b. Level of Consciousness Questions (month/age)	0 = Both answers correct 1 = One answer correct 2 = Neither answer correct		0 = Both answers correct 1 = One answer correct 2 = Neither answer correct
1c. Level of Consciousness Commands (closing eyes/hand grip)	0 = Performs both tasks correctly 1 = Performs one task correctly 2 = Performs neither task correctly		0 = Performs both tasks correctly 1 = Performs one task correctly 2 = Performs neither task correctly
2. Best Gaze	0 = Normal 1 = Partial gaze palsy 2 = Total gaze palsy		0 = Normal 1 = Partial gaze palsy 2 = Total gaze palsy
3. Visual	0 = No visual loss 1 = Partial hemianopia 2 = Complete hemianopia 3 = Bilateral hemianopia (blind including cortical blindness)		0 = No visual loss 1 = Partial hemianopia 2 = Complete hemianopia 3 = Bilateral hemianopia (blind including cortical blindness)
4. Facial Palsy	0 = Normal 1 = Minor paralysis 2 = Partial paralysis 3 = Complete paralysis	0 = Normal 1 = Minor paralysis 2 = Partial paralysis 3 = Complete paralysis	
5. Motor Arm (10 s) 5a—left 5b—right	0 = No drift 1 = Drift 2 = Some effort against gravity 3 = No effort against gravity, limb falls 4 = No movement UN = Amputation or joint fusion	0 = No drift 1 = Drift 2 = Some effort against gravity 3 = No effort against gravity, limb falls 4 = No movement UN = Amputation or joint fusion	0 = No drift 1 = Drift 2 = Some effort against gravity 3 = No effort against gravity, limb falls 4 = No movement UN = Amputation or joint fusion
6. Motor Leg (5 s) 6a—left 6b—right	0 = No drift 1 = Drift 2 = Some effort against gravity 3 = No effort against gravity limb falls 4 = No movement UN = Amputation or joint fusion	0 = No drift 1 = Drift 2 = Some effort against gravity 3 = No effort against gravity, limb falls 4 = No movement. UN = Amputation or joint fusion	0 = No drift 1 = Drift 2 = Some effort against gravity 3 = No effort against gravity, limb falls 4 = No movement UN = Amputation or joint fusion
7. Limb Ataxia	0 = Absent 1 = Present in one limb 2 = Present in two limbs UN = Amputation or joint fusion		
8. Sensory	0 = Normal 1 = Mild-to-moderate sensory loss 2 = Severe-to-total sensory loss	0 = Normal 1 = Mild-to-moderate sensory loss 2 = Severe-to-total sensory loss	0 = Normal 1 = Mild-to-moderate sensory loss 2 = Severe-to-total sensory loss
9. Best Language	0 = No aphasia 1 = Mild-to-moderate aphasia 2 = Severe aphasia 3 = Mute, global aphasia	0 = No aphasia 1 = Mild-to-moderate aphasia 2 = Severe aphasia 3 = Mute, global aphasia	0 = No aphasia 1 = Mild-to-moderate aphasia 2 = Severe aphasia 3 = Mute, global aphasia
10. Dysarthria	0 = Normal 1 = Mild-to-moderate dysarthria 2 = Severe dysarthria UN = Intubated or other physical barrier	0 = Normal 1 = Mild-to-moderate dysarthria 2 = Severe dysarthria UN = Intubated or other physical barrier	
11. Extinction and Inattention (formerly Neglect)	0 = No abnormality 1 = Mild 2 = Severe		0 = No abnormality 1 = Mild 2 = Severe

**Table 3:** Selected rating scales [42].

Scale	Rating System
Glasgow Coma Score	Eye opening Spontaneous (4) Response to verbal command (3) Response to pain (2) No eye opening (1) Verbal response (best) Oriented (5) Confused (4) Inappropriate words (3) Incomprehensible sounds (2) No verbal response (1) Motor response (best) Obeys commands (6) Localizing response to pain (5) Withdrawal response to pain (4) Flexion to pain (3) Extension to pain (2) No motor response (1)
Hunt and Hess	Asymptomatic or mild headache and slight nuchal rigidity (1) Severe headache, stiff neck, no neurological deficit except cranial nerve palsy (2) Drowsy or confused, mild focal neurological deficit (3) Stuporous, moderate, or severe hemiparesis (4) Coma, decerebrate posture (5)
Modified Rankin Scale	No symptoms (0) No significant disability despite symptoms: able to carry out all usual duties and activities (1) Slight disability: unable to carry out all previous activities, but able to look after own affairs without assistance (2) Moderate disability: requiring some help but able to walk without assistance (3) Moderately severe disability: unable to walk without assistance and unable to attend to own bodily needs without assistance (4) Severe disability: bedridden, incontinent, and requiring constant nursing care and attention (5) Dead (6)

## Data Availability

Not applicable.
